# Supplementary material for: Exploring service users experiences of remotely delivered CBT interventions in primary care during COVID-19: An interpretative phenomenological analysis
Source: PLoS One. 2023 Jan 6;18(1):e0279263. doi: 10.1371/journal.pone.0279263 (PMC9821471; doi:10.1371/journal.pone.0279263)
Supplement: S1 Table — (DOCX) [file pone.0279263.s001.docx]

|  | **Participants** | | | | | | | | |
| --- | --- | --- | --- | --- | --- | --- | --- | --- | --- |
| **Superordinate and subordinate themes** | **Jack** | **Fran** | **Sophie** | **Lisa** | **Beth** | **Kate** | **Nick** | **Polly** | **Adam** |
| **1. Orientating to treatment** |  |  |  |  |  |  |  |  |  |
| - 1. Expectations | √ | √ | √ |  |  | √ | √ | √ | √ |
| - 1. Role of GPs | √ | √ | √ |  |  | √ | √ | √ | √ |
| 1.3 Improving access | √ | √ |  | √ | √ | √ | √ | √ |  |
| **2. Treatment features** |  |  |  |  |  |  |  |  |  |
| 2.1 The helpfulness of CBT | √ | √ | √ | √ |  | √ | √ | √ | √ |
| 2.2 Remote delivery: benefits and challenges | √ | √ | √ | √ | √ | √ | √ | √ | √ |
| **3. Change enablers** |  |  |  |  |  |  |  |  |  |
| 3.1 Adapted material | √ | √ | √ | √ | √ | √ | √ | √ | √ |
| 3.2 Therapeutic relationship | √ | √ | √ | √ | √ | √ | √ |  | √ |
| 3.3 Taking responsibility for change | √ | √ |  |  | √ | √ |  | √ | √ |
| **4 Impact** |  |  |  |  |  |  |  |  |  |
| 4.1 Coping in a different way | √ | √ | √ | √ | √ | √ | √ | √ | √ |
| 4.2 Awareness and understanding | √ | √ | √ |  | √ | √ | √ |  | √ |
| 4.3 Valuing the Self | √ | √ |  |  | √ | √ |  | √ | √ |
| 4.4 Feeling grateful | √ | √ | √ | √ | √ | √ | √ | √ | √ |

**S1 Table. Participants’ contributions to the themes**
