# Supplementary material for: Exploring service users experiences of remotely delivered CBT interventions in primary care during COVID-19: An interpretative phenomenological analysis
Source: PLoS One. 2023 Jan 6;18(1):e0279263. doi: 10.1371/journal.pone.0279263 (PMC9821471; doi:10.1371/journal.pone.0279263)
Supplement: S1 File — (DOCX) [file pone.0279263.s002.docx]

**Excerpt of Transcript and IPA Analysis**

| **Emergent themes** | **Transcript**  Interview 9 - January 2021 - Video call  Interview Duration: 1 hour  I: Interviewer P: Participant | **Exploratory comments**  Descriptive comments: Roman text.  Linguistic comments: *Italic text*.  Conceptual: Underlined text. |
| --- | --- | --- |
| Straightforward access  Challenges remote delivery- video  Medium preference  ‘real’ F2F contact – nurturing  Being in the same room, read body language to connect  Impact of Covid on homework.  Reading more beneficial than homework  Relevant and powerful tool –  Personalisation  Initial feelings (fear and pressure) and expectations  Mixed initial feelings (fear, excitement, relief)  Impact of therapy - Coping with emotions | I: in terms of accessing the service during Covid-19 restrictions, did you experience any barriers?  P: no, it was quite straightforward I mean it was similar to what I am doing with work, sometimes the connection could be a bit poor, it was usually from *the psychologist* and there was one or two issues sometimes just with the connection but when the connection would fail, it would be a phone call, we would continue with a phone call which wasn’t ideal, I always preferred face-to face but there is no issues with the Covid thing, it worked quite straightforward like this, so no, there was no issue.  I: ok, so apart from connection issues you did not experience any barriers  P: yeah yeah  I: you mentioned that you preferred video call over phone call, tell me more about that  P9: I just I think it is more a nurturing element to the therapy when you can actually see the person, it is a human contact whereas telephone is just a voice over the phone so if you can’t physically be in the same room so this is the next best thing, you can read their body language and see how they are responding to what you are saying, you can’t do that with the phone. It’s more intimate, personal, so I just preferred.  …….  I: what about doing the exercise part?  P: apart from the restrictions which did not allow me to do things, I found the reading more beneficial than the actual exercises but there was *one [showing their index]*exercise that was *excellent*, that helped me cope with distress tolerance action plan I actually have that up in my bedroom wall so it’s there every day, the distress tolerance action plan made a lot of sense and I am able to use it at any time I am just feeling down or a bit self-destructive, that was a very powerful useful tool the other ones were a bit more writing down thoughts diary and daily planners, those things are really.. it just doesn’t really work for me, I know everyone is different so, I am not saying that they are bad exercises, I just think that for me weren’t a preference, I didn’t find it beneficial, maybe more time-consuming than anything but it was 50-50%.  …  I: what your experience of being in therapy?  P: it was all new, there was kind of fear and there was pressure because I thought, ‘if this does not work then I am *fu*d’* excuse the language – *haha [nervous laugh*] - people said this is supposed to work if it doesn’t then what do I do? there was probably I lot of fear but maybe if it fails I am in a lot of trouble because ‘what do I do?’ the experience at the start there was quite a lot of fear there because it was so new and I was putting lot of emphasis on that working maybe that distracted me from the first 2 months of it, I was focusing on the wrong things as supposed to the work so I was quite scared at the start I was excited… I was really relieved when the NHS called me it was excitement and relief but when the date was getting closer for the first session when she spoke to me on the phone it kind of change to a bit more fear.  I: what helped with that fear?  P: when I started to notice that it was working, we were working on emotions, that was obviously very convenient because I had these feelings of fear and I was reading about fear, sadness and anger and frustration and how to process and deal with them and live with them, the therapy helped with that feeling of fear, it was just convenient, I was lucky I was studying emotions at the start it was working with emotions, the therapy helped with that. | Got used to remote way of communicating – is it becoming familiar? *P’s tone of voice emphasised their frustration caused by therapist’ Wi-Fi issues.*  Why does P prefer F2F? What is the real experience and impact of F2F vs remote delivery?  Being in the same room seems to make the relationship more ‘human’. Is this aspect replaceable in remote delivery ? if yes, how?  P found the reading very helpful. Why so? How did the reading impact P’s recovery?  *P’s gesture and tone of voice emphasised the relevance and usefulness of that exercise*. Good example of how important is to tailor material and exercise to individual needs. Importance of having a visually available action plan  how important is to have a visual reminder/prompt?  Therapy as something new, last resort and possible failure for P. *P’s nervous laugh and language used highlighted the initial powerful emotional state and expectation at the start of therapy.* Are patients’ initial feelings and expectations overlooked in first sessions by clinicians?  Reading and learning about emotions helped P to deal with their initial fear. Material and psychoeducation can lead to immediate changes in therapy.  Importance of applying what recently learnt in therapy and beyond. |
