# Supplementary material for: Exploring service users experiences of remotely delivered CBT interventions in primary care during COVID-19: An interpretative phenomenological analysis
Source: PLoS One. 2023 Jan 6;18(1):e0279263. doi: 10.1371/journal.pone.0279263 (PMC9821471; doi:10.1371/journal.pone.0279263)
Supplement: S2 File — (DOCX) [file pone.0279263.s003.docx]

**Extracts of Reflective Journal – IPA**

I feel very apprehensive and nervous today as I am going to conduct my first interview for the empirical study. I am also curious about how it is going to be via video call. Will I be able to stay calm and connect with the participant to elicit personal material? maybe I just need to see this interview as a learning experience…

…It went much better than expected. Participant number 1 talked and shared a lot. Although I realised how difficult it is to ask ‘neutral’ and research-oriented questions… at times I had to remind myself of the interview aim, it wasn’t an assessment session and I had to stick to the interview schedule and prompts…I am not sure I asked the questions in the best way possible.. I need to discuss this in supervision.

…I am so glad I discussed my doubts and insecurities about my first interview in supervision. My supervisor read my transcript and gave me helpful feedback about how to keep an open, curious and research-oriented stance in semi-structured interviews…I will apply these suggestions in my next interview.

…initially I felt frustrated as this participant did not really articulate their responses much. I tried to ask open questions and prompts but no change. All I got for most of the interview was “everything was good, fine”. This was the shortest interview so far and affected my confidence. The fact that it was over the phone, I think, did not facilitate the conversation with this person… I will talk about this in supervision…I guess every participant is different and maybe there is a cultural aspect to take into account …I might hold unrealistic expectations on ‘how interviews should be’ in qualitative research….something to discuss in supervision…

Listening to all these positive experiences of psychological interventions in Primary Mental Health Care helped me to reconnect with the purpose of the research after finding the recruitment phase so long and uncertain due to Covid-19 pandemic and recruitment suspension. It also brought back memories from my experience in IAPT..I feel really lucky to be getting an insight into people’s experience of therapy. It’s something I also wondered in my clinical practice in previous jobs (e.g. PWP) and as a Trainee Clinical Psychologist. I extremely value patients feedback and insights…

..whilst transcribing this interview, I felt that this participant really emphasised both the role of their therapeutic relationship and material in their journey. I started to reflect on how these aspects are/were present in my clinical practice and service where I work/worked…

This participant mentioned how lucky and grateful they felt as they were able to access psychological interventions through the NHS, a public and free service. This account really resonated with me as, unfortunately, provision of psychological therapies in Primary Care services in other country (e.g. Italy) is absent…

During IPA analysis and whilst writing the results I was particularly anxious as I want to make sure that this research represents each participant voice and experience as best and accurate as possible….I found Smith et al. book really helpful for novice IPA researchers like myself..

I am trying to generate subordinate and superordinate themes for the first time and it feels overwhelming.. it is a lot of data to represent in a few themes and in a coherent interpretation. I am finding myself worrying about my interpretations…are they good enough? I realised and accepted that in qualitative studies, researchers’ interpretations are some of the possible understanding of the data…

I am glad that my academic and field supervisor are going to code some transcripts independently. I feel less alone and lost in this process…Discussions with my supervisor helped me finalising the themes and made me reflect on my interpretations and on the IPA distinctive characteristics.

I have interviewed my last participant and I feel so relieved. I also feel that my interview skills improved a lot throughout this research. I feel more confident in asking open and neutral questions. I became really absorbed in what the participant was saying and less worried about what to ask next. The conversation flew much better…

While reading this diary near the end of this project I realised that my position in relation to some topics changed and I became more aware of some issues around access and orientating to treatment after listening to service users’ experiences…

I regret not planning to check the themes with service users. I will highlight this as a limitation in my study and will definitely include this step and qualitative control measure in my next qualitative research.
