# Supplementary material for: Exploring service users experiences of remotely delivered CBT interventions in primary care during COVID-19: An interpretative phenomenological analysis
Source: PLoS One. 2023 Jan 6;18(1):e0279263. doi: 10.1371/journal.pone.0279263 (PMC9821471; doi:10.1371/journal.pone.0279263)
Supplement: S4 File — (PDF) [file pone.0279263.s005.pdf]

IRAS ID: 270413

INTERVIEW SCHEDULE

Accessing the service

- e.g. what was your experience of accessing the service?
- What barriers did you experience when accessing treatment delivered remotely during the Covid-19 restrictions?

Prompts: tell me more about the referral process as you experienced it; tell me what was helpful/unhelpful about accessing the service

Assessment process

- e.g. what was your experience of the assessment process?

Prompts: tell me more about what the assessment involved; tell me about any aspects of the assessment process that you would have liked to see proceed differently

Engaging with the service

- e.g. How was your engagement with the service?
- How did you experience psychological interventions in primary care during the Covid-19 restrictions?
- What barriers did you experience when engaging in treatment delivered remotely during the Covid-19 restrictions?

Prompts: tell me more about what helped/hindered your engagement with the service; tell me more about your experience of engaging in psychological treatment

Completing treatment

- e.g. How would you describe your experience of completing treatment and be discharged?

Prompts: tell me more about what receiving treatment was like; tell me more about what completing treatment was like

Treatment impact

e.g. How did the treatment affect you?

Prompts: tell me more about what you found helpful/unhelpful about the treatment

Interview Closure

Is there anything I did not ask and you would like to add?

How have you found this interview?
