# Supplementary material for: Exploring service users experiences of remotely delivered CBT interventions in primary care during COVID-19: An interpretative phenomenological analysis
Source: PLoS One. 2023 Jan 6;18(1):e0279263. doi: 10.1371/journal.pone.0279263 (PMC9821471; doi:10.1371/journal.pone.0279263)
Supplement: Attachment — Submitted filename: Response to reviewers.docx [file pone.0279263.s007.docx]

**Response to Reviewers**

**1. Journal Requirements**

Please review your reference list to ensure that it is complete and correct. If you have cited papers that have been retracted, please include the rationale for doing so in the manuscript text, or remove these references and replace them with relevant current references. Any changes to the reference list should be mentioned in the rebuttal letter that accompanies your revised manuscript. If you need to cite a retracted article, indicate the article’s retracted status in the References list and also include a citation and full reference for the retraction notice.

**Thank you for highlighting this regarding the reference list. Following the reviewers comments on the first submission, four papers have been retracted as the section of the manuscript comparing IPA with other methodologies was deleted. The references removed were:**

1. Starks H, Brown Trinidad S. Choose your method: A comparison of phenomenology, discourse analysis, and grounded theory. Qualitative health research. 2007 Dec;17(10):1372-80.
2. Smith JA. Reflecting on the development of interpretative phenomenological analysis and its contribution to qualitative research in psychology. Qualitative research in psychology. 2004 Jan 1;1(1):39-54.
3. Braun V, Clarke V. Using thematic analysis in psychology. Qualitative research in psychology. 2006 Jan 1;3(2):77-101.
4. Urquhart C. Grounded theory for qualitative research: A practical guide. Sage; 2012 Nov 16.

**Following the reviewers comments on the first submission, three papers have been added to elaborate on the points raised in the manuscript. These references were:**

1. Gratzer D, Khalid-Khan F. Internet-delivered cognitive behavioural therapy in the treatment of psychiatric illness. Cmaj. 2016 Mar 1;188(4):263-72.
2. Schröder J, Berger T, Westermann S, Klein JP, Moritz S. Internet interventions for depression: new developments. Dialogues in clinical neuroscience. 2022 Apr 1.
3. Beattie A, Shaw A, Kaur S, Kessler D. Primary‐care patients’ expectations and experiences of online cognitive behavioural therapy for depression: a qualitative study. Health expectations. 2009 Mar;12(1):45-59.

**2. Reviewer #1**

Thanks for addressing my concerns the details are provided are fine. Please note one reference (2009) can change. Very best wishes for your future research.

**Thank you for your helpful comments and wishes.**

**3. Reviewer #2**

**Thank you for your comments. We have incorporated all your suggestions.**

Methods

Thank you for addressing my comment about the Approach section. I like how this is looking, but I would still make sure you have this section focussed more on the Approach, and less on the IPA analysis. This section only needs to focus on the methodology (e.g., phenomenology e.c.t), rather than how it is actually applied. As so, you can probably remove the content from 140 – 146. I also wouldn’t put the approach section in place of the analysis of data section. I think both are still needed here, especially as the analysis of data section will allow you to go a bit more into depth about the six steps and how you applied them specifically to your study.
I would maybe also include a methods section specifically looking at your participants as this information doesn’t really count as ‘results’. I feel it would also likely fit into your setting, eligibility and recruitment section too.
There are a few nice examples of methods in these studies: <https://doi.org/10.1080/0963823031000118203>; <https://doi.org/10.1371/journal.pone.0265542>; <https://doi.org/10.2196/mental.9934>

**Thank you for your comments and examples provided.**

**A section focused on the IPA approach and a section focused on the Data analysis are now both present and distinct. Content from 140 – 146 has been moved to the Data analysis section. Information about research participants is now available in Setting, eligibility criteria, recruitment and participants.**

Results

While this is more a recommendation than a must, but with the removal of the table showing participant’s contributions to themes, I would create a new table/figure which simply illustrates your theme structure. It does not need to be comprehensive as to include participant’s alignment with certain themes, but it would be useful to see your final super-ordinate and sub-ordinate themes at a glance. You could also number them in alignment with the section (see my comment on formatting). The first two DOI’s attached in my comment on methods also have tables like these which might be useful to view.

**Thank you for your comments and examples provided. Figure 1 has been added to the Result section and illustrates the theme structure.**

Formatting

I would say that if you’re going to number your main headings (e.g., 2. Methods) to also number your sub-headings (2.1. IPA Approach) just for the sake of consistency throughout. You could also do this with your themes (e.g., 3.2.1: The helpfulness of CBT).

**Thank you for highlighting this. The manuscript sections have been formatted as suggested.**

**We very much hope that we have addressed all the points raised and that and the edited manuscript meets the journal standards. We look forward to hearing from you.**
